# New systematic study approach of green synthesis CdS thin film via Salvia dye

**DOI:** 10.1038/s41598-022-16733-y

**Published:** 2022-07-22

**Authors:** A. S. Najm, Hasanain Salah Naeem, Khalid O. Alabboodi, Siti Aishah Hasbullah, Hiba Ali Hasan, Araa Mebdir Holi, Asla Abdullah AL-Zahrani, K. Sopian, Badariah Bais, Hasan Sh. Majdi, Abbas J. Sultan

**Affiliations:** 1 Department of Electrical, Electronics and System, FKAB, Universiti Kebangsaan Malaysia (UKM), 43600 Bangi, Selangor Malaysia; 2 Al-Muthanna University, Al-Resala, Samawah, Al-Muthanna Iraq; 3 Department of Chemical Engineering and Petroleum Industries, Al-Mustaqbal University College, Babylon, 51001 Iraq; 4 School of Chemical Sciences and Food Technology, Faculty of Science and Technology, Universiti Kebangsaan Malaysia (UKM), 43600 Bangi, Selangor Malaysia; 5 Department of Pharmacognosy and Medicinal Plants, College of Pharmacy, Mustansiriyah University, Baghdad, Iraq; 6 Department of Physics, College of Education, University of Al-Qadisiyah, Al-Diwaniyah, Al-Qadisiyah 58002 Iraq; 7 Imam Abdulrahman-Bin Fiasal University, Eastern Region, Dammam, Saudi Arabia; 8 Solar Energy Research Institute (SERI), Universiti Kebangsaan Malaysia (UKM), 43600 Bangi, Selangor Malaysia; 9 Department of Chemical Engineering, University of Technology, Baghdad, Iraq

**Keywords:** Materials chemistry, Chemical physics

## Abstract

In this study, we aimed to increase the knowledge regarding the response mechanisms which were associated with the formation of CdS thin films. CdS thin film remains the most appealing alternative for many researchers, as it has been a capable buffer material for effect in film based polycrystalline solar cells (CdTe, CIGSe, CZTS). The Linker Assisted and Chemical Bath Deposition (LA-CBD) technique, which combines the Linker Assisted (LA) technique and the chemical bath deposition (CBD) method for forming high quality CdS thin film, was presented as an efficient and novel hybrid sensitization technique. CdS films were bound to soda lime with the help of electrostatic forces, which led to the formation of the intermediate complexes [Cd (NH_3_)_4_]^2+^ that helped in the collision of these complexes with a soda lime slide. Salvia dye and as a linker molecule 3-Mercaptopropionic acid (MPA) was used in the one step fabrication technique. Optical results showed that the bandgap varied in the range of (2.50 to 2.17) eV. Morphological properties showed a homogeneous distribution of the particles that aspherical in shape in the CdS + MPA + Salvia dye films. This technique significantly affected on the electrical characterizations of CdS films after the annealing process. The CdS + Ag + MPA + Salvia dye films showed the maximum carrier concentration and minimum resistivity, as 5.64 × 10 ^18^ cm^−3^ and 0.83 Ω cm respectively.

## Introduction

**Figure 1:**
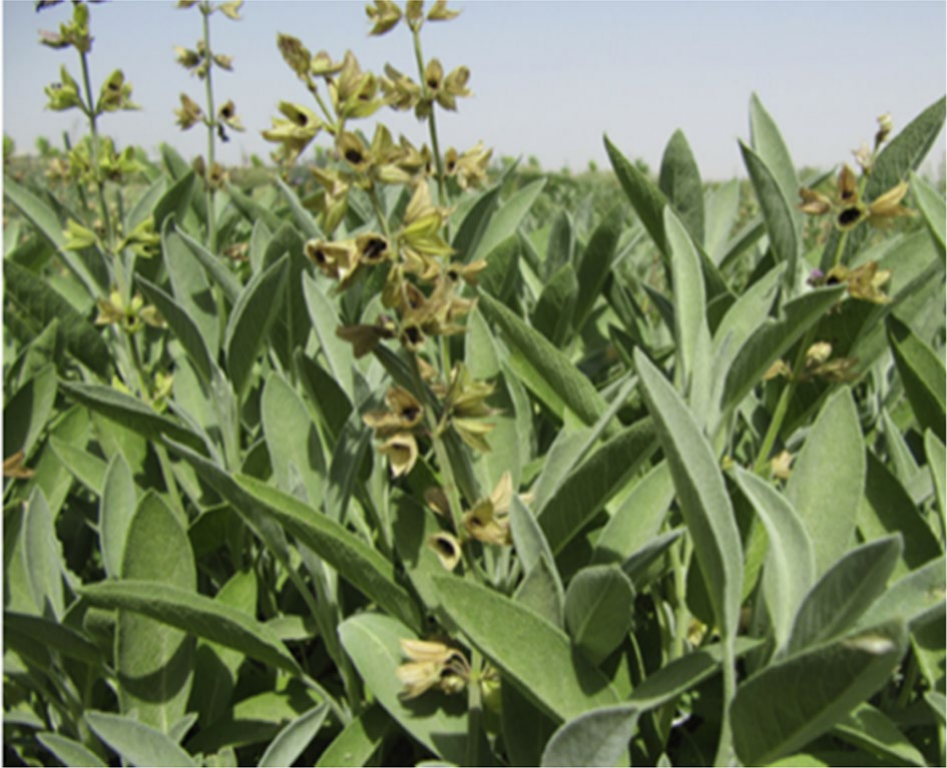
*Salvia officinalis* L.^8^.

The transfer from computational approaches to experiential manners of real catalysts is still a challenge. Metal nanoparticles in solution, due to their high dispersion, appear to agglomerate and coagulate spontaneously, and must therefore be stabilized^1^. Recently, great concerns have emerged over nanomaterials’ potential adverse impacts on the eco-environment due to the increased use of theirs^2^. Since then, the ongoing environmental influence of nanomaterials is currently insufficiently researched and discussed, including how to best validate this effect^3^. Nanoparticles can be made in a green way and used for a variety of antibacterial and anti-cancer applications^4^. During the nanoparticle preparation process, natural compounds are employed to decrease metal salts, and no other reducing agents or stabilizing agents are applied. The nanoparticles that were created have excellent biological characteristics^5^. Fierascu et al. synthesized gold nanoparticles from Salvia officinalis (SO) extract^6^. While (Karel Sehnal 2019) evaluated the effect of varying concentrations of Ag NPs on maize germinated plants utilizing a green approach (using sage extract) in comparison to Ag(I) ions (*Zea mays*)^7^. *Salvia officinalis* L. (common sage) is an aromatic perennial evergreen subshrub, and is native to the Mediterranean region, Southeast Africa, and Central and South America, Fig. 1.

Although the ability of some sage species to biosynthesize compounds is of interest to the food and pharmaceutical industries, practically all investigations in the literature to our knowledge are limited to a few papers for utilizing as a capping agent in nanoparticle synthesis. Furthermore, no information about its performance comparison is provided in the literature. Some earlier reports showed that, both CdS and HgCdTe film are II-VI group semiconductors, and have a great potential in photodetection^9,10^. More specifically, the semiconductor molecules such as CdS thin film due to it is consider as a promising buffer layer, which could be used as the conventional n-type heterojunction partners in the existing and the new thin-film PV devices, owing to their direct bandgap transition (E_g_ ~ 2.4 eV), transparency, n-type conductivity and a direct bandgap transition with a high electron affinity (4.2 eV)^11^. Although it is considering as toxic material, but the quantity we used within the fabrication of solar cell as a buffer layer is around 100 nm, which is very tiny. In addition to that, for more improving the properties of the CdS thin films, we took advantage of the QDs and aimed to stabilise the surfaces of the CdS nanocrystal thin films by using proper organic molecules called capping agents. These could be used during the synthesis and bind to the particle surfaces, thus decreasing the growth of the particles and preventing aggregation. Without forgetting how this synthesis can impact in term of the sustainable environment. In an earlier study, Kovalenko et al. noted that when they used molecular metal chalcogenide surface ligands near the QDs, they could preserve the size-dependent optical absorption properties of the molecules, while the electron mobility was significantly improved^12^. Yu et al. proposed the in-situ Linker-Assisted Chemical Bath Deposition (LACBD) technique for fabricating the photostable CdSe/CdS QD-sensitised TiO_2_ surfaces by using a bi-functional modifier, i.e., Thioglycolic Acid (TGA)^13^. The QDs which were synthesised using this technique were smaller in size and showed a narrow size distribution compared to the traditional CBD technique owing to the stabilising nature of the TGA. Till date, thiols were considered the best ligands which helped in controlling the growth and nucleation of the II–VI semiconductor nanocrystals^14^. Out of the different thiol-based ligands, the ligands with a mercapto-group and one carboxyl group that is connected with an alkyl chain, have been generally used. 3-MercaptoPropionic Acid (MPA) is seen to be an organic molecule, with 2 functional groups. The coordination between one or both of these functional groups and the nanoparticle surfaces show the two advantages, i.e., (1) Passivation of the dangling bonds to nanoparticle surface; and (2) Protection of nanoparticles and their prevention from attracting one another, which inhibits aggregation. MPA is a popular ligand since its usage leads to a low-density of mid-gap state, which allows the collection of charge carriers over longer distances outside their depletion region^15^.

With regards to the use of CdS films as a buffer layer, it was stated that these films must be very thin, which helps in maintaining a low-series resistance and high photon transmission. This allowed optimised minority carrier transport. However, if the CdS films were thick, it induced a Schottky barrier effect and improved the minority carrier transportation^16^. Doping semiconductors after incorporating acceptors or donors into the crystal lattice was a conventional method for reducing electrical resistivity^17^. As doping grows in crystalline cadmium sulphide lattice, the depletion area declines consequently carriers’ concentration and mobility show improvement, whereas the value of the semiconductor working function decreases. This doping could be carried out using an in-situ chemical process during the growth, wherein we added a specific volume of the salt solution of doping atoms to the reaction solution, without harming the crystal lattice structure. Recently, many studies have attempted to investigate and develop a modified CdS bandgap by determining point defects and doping processes for increasing the absorption of the incident light. The popular atoms used for doping the CdS films included indium, tin, copper, gallium, aluminium, and magnesium^17–22^. Silver (Ag) was a Group I element, which acted as the donor dopant of (II–VI) semiconductors and improved their electrical properties. This doping could be incorporated in the CdS nanoparticles without affecting their inherent crystal lattice structure. The potential difference noted between the conduction band of the CdS and the Fermi level of Ag helped in transferring electrons between the doped material and the semiconductor matrix^23^. Taur et al. analysed the impact of annealing on the physical–chemical and optoelectronic properties and reported I–V responses from the growing and annealed of thin films showed an improvement from 72 to 96% of photosensitivity after illumination to 100 mW/cm^2^ light source^24^. Whereas Ferrá-González et al. noticed that the bandgap in energy and roughness is considerably increasing with the concentration of silver, to the point that cadmium is depleted and stopped being substituted, silver sulphide (Ag_2_S) is starting to form at this point, the bandgap and film roughness is beginning to decline with the rise in the concentration of AgNO_3_^25^. Furthermore, Flores-Pacheco et al. also indicate that the existence of a polycrystalline structure for the Ag^+^ doped influenced quantum confinement with a decrease in the average particle size from 5.46 to 4.12 nm, resulting in higher energy emissions due to the drop in particle size under the efficient CdS exciton Bohr radius^26^. Sergio et al. noted that the roughness and the bandgap energy increased with an increasing Ag concentration, up to the point of Cd depletion.

Though some studies investigated the doping by Ag atoms and noted that the surface of all doped films showed the presence of aggregates, which was generally noted when the materials were grown using the CBD technique.

Hence, this study aims to develop a new approach for the synthesis of CdS thin film by combining the Linker-Assisted CBD (LACBD) technique with Salvia dye, MPA, and Ag doping via using the CBD method for forming high-quality CdS thin films. We also described the mechanism which inhibited the formation of the thin CdS films. In theory, CdS thin-film semiconductor can be achieved from the chemical bath deposition method through the direct reaction of [Cd^+2^] and [S^−2^] precursor species in solution. For LACBD, our new method offers the great benefit being able to be performed in situ by simply addition of Salvia dye, MPA and Ag doping after a specific time to control the growth of reaction. Here, Salvia dye for as a natural capping agent is employed here as the first time, as a stabilizer for controlling the formation and connecting them to the substrate.

## Experimental details

### CdS thin film deposition by the LACBD process

Many different types of precursors and syntheses can be used depending on the expected results. We initially prepared 3 stock solutions, firstly; the fresh Sage Plant (from Arabic shop in Malaysia) was washed repeatedly with water to eliminate the dust and then allowed to dry at room temperature in a shade until became crisp. After being dried, they were crushed in a home mixer to make them into powder, then dissolve in DI water under stirring in the hot plate at 360 rpm, and 25 °C for 24 h. The extract solution was then filtered and used as a Salvia dye. While the second stock solution is for preparing the Ag stock solution, silver nitrate (AgNO_3_) dissolved into deionised water (50 ml) to yield a 0.01 M silver nitrate solution. The last stock solution is for MPA (0.212 g, 2 mmol) in a methanol-deionised water mixture (10 ml methanol mixed with 3 ml water). The pH has been adjusted to be 10 by using KOH base^27^.

**Figure 2:**
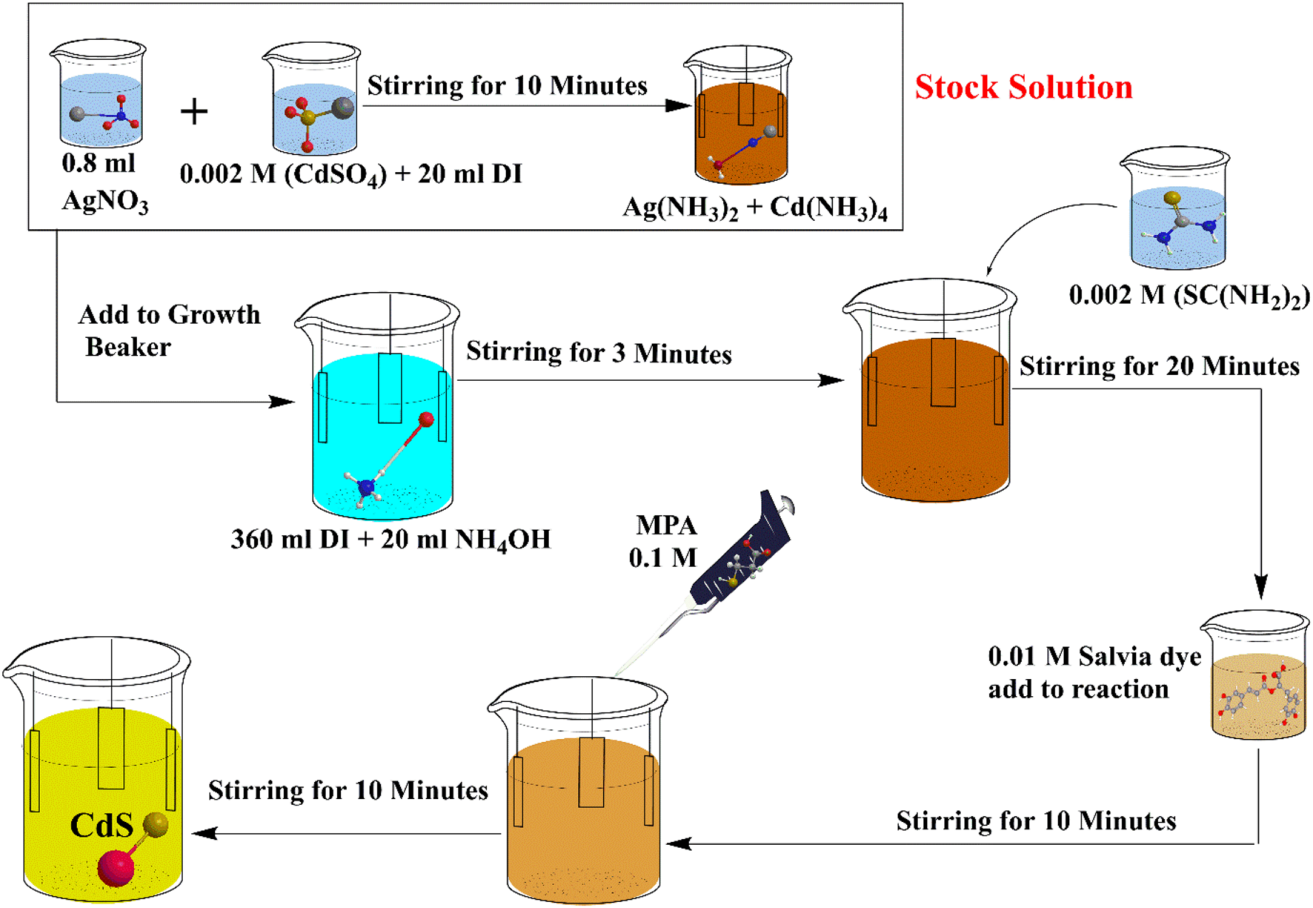
Synthesis of CdS capped with (Ag + MPA + Salvia dye) by CBD process.

CdS thin film was synthesised in accordance with the Yulisa et al.^28^ with modification. In this process, we ultrasonically cleaned and degreased soda lime glass slide substrate (25 mm × 25 mm). The chemical bath was prepared using DI water and ammonium hydroxide solution by volume (10:1 v/v). Thiourea (0.002 M) and cadmium sulphate (0.002 M) were used as the source of sulphur and cadmium salts, respectively. Two stages of experiments were used in this methodology. Stage 1 involved CdS synthesis using two approaches, traditional CdS thin films and Salvia dye sensitised CdS. Stage 2 involved the optimisation of CdS via the hybrid process which included the mixing of Salvia dye + AgNO_3_, Salvia dye + MPA, and Salvia dye + AgNO_3_ + MPA all in the same experiment. According to our methodology, we added Salvia dye after 20 min, as rendering to Sandoval and Ramírez who investigated the early growth stages of the CdS thin films during their chemical deposition. They noted that the growth took place at the deposition times ranging between 15 and 18 min, which yielded a dense and compact CdS inner layer^29^. Hence, for optimising the growth mechanisms of the CdS thin films, Salvia dye has been added to the chemical reaction after 20 min. The summary of synthesis of the CdS thin film by different concept behaviour has been shown in Table 1. Whereas Fig. 2 shows the synthesis of CdS thin film at mix all case.

**Table 1:** CdS-CBD synthesis cases.

Synthesis	Chemical quantity	Reaction condition	Description	Appearance
Basic CdS	DI water: 360 ml CdSO_4_: 0.002 M SC(NH_2_)_2_: 0.001 to 0.05 M NH_4_OH: 3.5 M, 40 ml	30 min, 80 °C	1. Initially, cadmium sulphate was added to the reaction beaker 2. Following three minutes, thiourea is added to the beaker 3. The reaction will take 30 min to complete	
Salvia dye capped CdS	DI water: 360 ml CdSO_4_: 0.002 M SC(NH_2_)_2_: 0.002 M NH_4_OH: 3.5 M, 40 ml Salvia dye: 0.01 M	30 min, 80 °C	1. Salvia dye also adds to CdS basic preparation after 20 min from the reaction started, in different concentrations 2. After 10 min the reaction has been completed and ready for characterizations	
Ag doped + Salvia dye capped CdS	DI water: 360 ml CdSO_4_: 0.002 M SC(NH_2_)_2_: 0.002 M NH_4_OH: 3.5 M, 40 ml AgNO_3_: 0.8 ml Salvia dye: 0.01 M	30 min, 80 °C	1. Ag% was added slowly to the Cd salt beaker, then in the ultrasonic bath for 10 min 2. After 20 min of starting the reaction, Salvia dye has been added 3. After 10 min the reaction has been completed and ready for characterizations	
MPA + Salvia dye capped CdS	DI water: 380 ml CdSO_4_: 0.002 M SC(NH_2_)_2_: 0.002 M NH_4_OH: 3.5 M, 20 ml MPA: 0.1 M Salvia dye: 0.01 M	40 min, 80 °C	1. After 20 min of starting reaction, Salvia dye has been added 2. After another 10 min, MPA has been added 3. After 10 min the reaction has been completed and ready for characterizations	
Ag + MPA + Salvia dye doped and capped CdS	DI water: 380 ml CdSO_4_: 0.002 M SC(NH_2_)_2_: 0.002 M NH_4_OH: 3.5 M, 20 ml AgNO_3_: 0.8 ml MPA: 0.1 M Salvia dye: 0.01 M	40 min, 80 °C	1. Ag% was added slowly to the Cd salt beaker, then in the ultrasonic bath for 10 min 2. After 20 min of starting the reaction, Salvia dye has been added 3. After another 10 min, MPA has been added 4. After 10 min the reaction has been completed and ready for characterizations

### Characterisation

Optical properties were measured from the wavelength range of 350–650 nm, with the help of a Lambda 950 UV/Vis/NIR spectrometer (Perkin-Elmer, USA). The respective spectrum was used to calculate the optical band gap of the films. The films’ structural characterizations were examined at room temperature, with the help of an AXS-D8 Advance Cu-Kα diffractometer (Bruker Corp., USA). We also studied the XRD patterns in a 2ϴ range, with a step size of 0.02°, which ranged between 10° and 80°, using the Cu-Kα radiation wavelength, λ, of 1.5408 Å. FEI Quanta 400F field emission scanning electron microscope (FESEM) equipped with Oxford- Instruments INCA 400 X-Max detector for energy-dispersive X-ray spectroscopy (EDX) measurement at × 300 magnification (spot size 1 mm × 1 mm) and an accelerating voltage of 20 kV. Lastly, the electrical characteristics of films were measured using a 0.57 T magnetic field and 45 nA probe current HMS ECOPIA 3000 Hall Effect measuring device. We applied the Ag paste procedure to make an ohmic contact by adding Ag dots in each four corners for our samples, then repeated each reading for each sample 10 times to increase credibility of our results.

## Results and discussion

### Optical properties analysis

**Figure 3:**
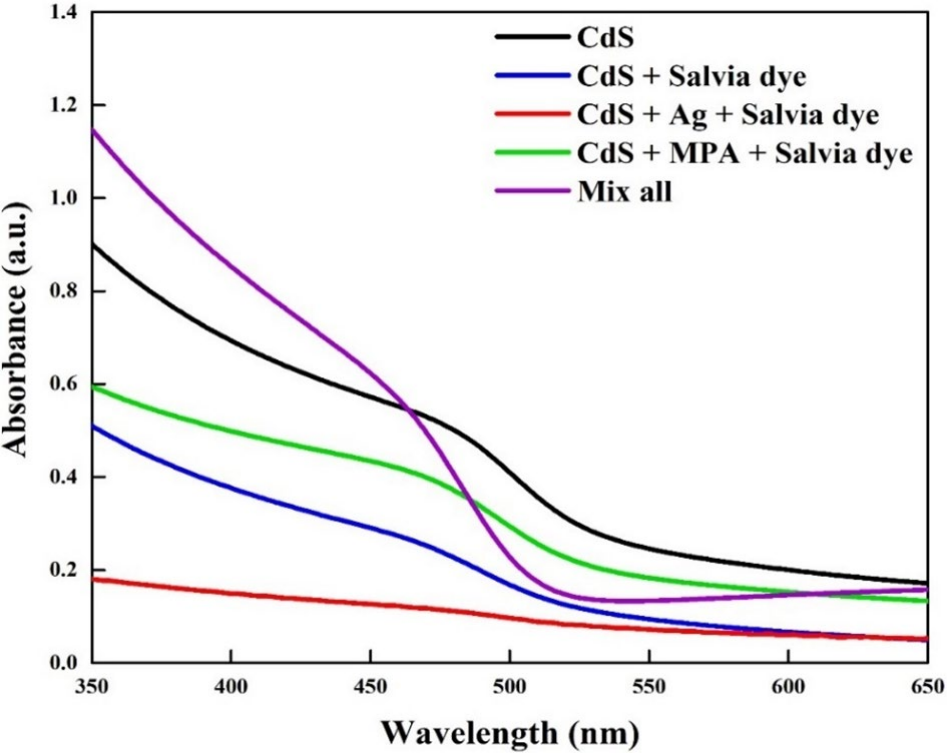
UV–Vis absorption spectra of the CdS thin films.

The relatively higher absorption of visible light by the films can offer a lot of information regarding the thin films^30^, Fig. 3.

The results demonstrated that, after Salvia dye is introduced, a decline in the CdS absorbance peak occurred due to the competition between the dye molecules and CdS that occurred within CdS structure^31^. While (CdS + Ag + Salvia dye) sample showed the lowest absorption spectra. This can be explained by mixing Ag with Salvia dye, which has the potential for ionic displacement and can create a higher number of lattice defects such as ionic vacancies, and so on. These defects function as trap centres and affect optical absorbance. Consequently, the formation of localized energy states at the band edge, the reduction in optical band intensity could be attributed to an optical defect or, more possibly, defect-induced band tailing^24^. The intensity peak corresponding to mixing all additives led to a slight blue shift for the absorption edge, possibly due to a decrease in the electron density inside the valence band. The combination between MPA and Salvia dye shows medium interactions among the adsorbed dye and the MPA molecules caused to reduce the aggregations of dyes, as a result, the observed broadening of the absorption spectral profile (widening of the full width and half maximum) can be attributed to this^31^. In other words, when the Salvia dye introducing to MPA, the absorbance was quenched. Results are supported by the study of Hassan et al., which found that the absorption intensity is quenched by competition between the two compounds on the surface^32^.

**Figure 4:**
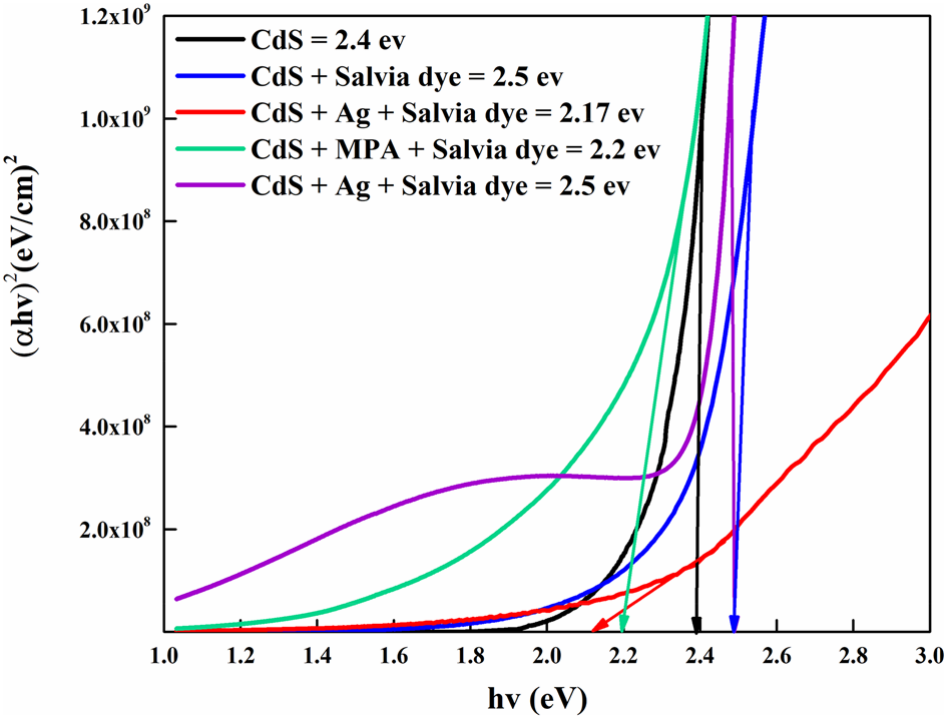
Variant of (*αhν*)^2^ with photon energy (*hν*) for CdS thin film.

The values of the optical bandgap are achieved through the dispersion relationship along with the essential absorption edge corresponding to the semiconductor direct bandgap by using Tauc’s plot^33^, Fig. 4. Optical bandgap (E_opt_) and optical absorption coefficient (α) are related in the transition direct semiconductor, as follows,^34^:

1 $$\alpha hv={B\left(hv-{E}_{g}\right)}^{1/2},$$

where *α* is the coefficient of absorption, *hv* is the photon energy, *E*_*g*_ is the direct band gap energy, *B* is a Boltzmann constant, and 0.5 is the value assuming for the direct bandgap nature of the material.

In terms of the absorption edge, the nature of the behaviour for bandgap energy has already been addressed. Both samples (CdS + Salvia dye) sample and (CdS + mix all) sample at 2.5 eV show quantum confinement induced by the limited particle size following a comparison with the bandgap value of bulk CdS at (2.4 eV)^35^. This quantum confinement effect is linked to changes in electrical characteristics caused by shifting the energy level positions of the conduction and valence bands to more negative and positive values, correspondingly. This redox potential shift favours electron transport pathways and boosts photoactivity^36^. This behaviour can be clarified by the assumption that the presence of Salvia dye and its interaction result in the formation of new molecular dipoles, which may result in less defects generated within the bandgap. For the (CdS + Ag + Salvia dye) and (CdS + MPA + Salvia dye) films, the incorporation of these doping agents, along with a substantial sulphur deficiency, would rise in donor levels in the CdS bandgap^37^. The donor levels degenerate and combine with the CdS conduction band when doping materials mixes, allowing the conduction band to expand into the forbidden region, decreasing the bandgap as 2.17 and 2.2 eV, respectively.

### Structural properties analysis by XRD

**Figure 5:**
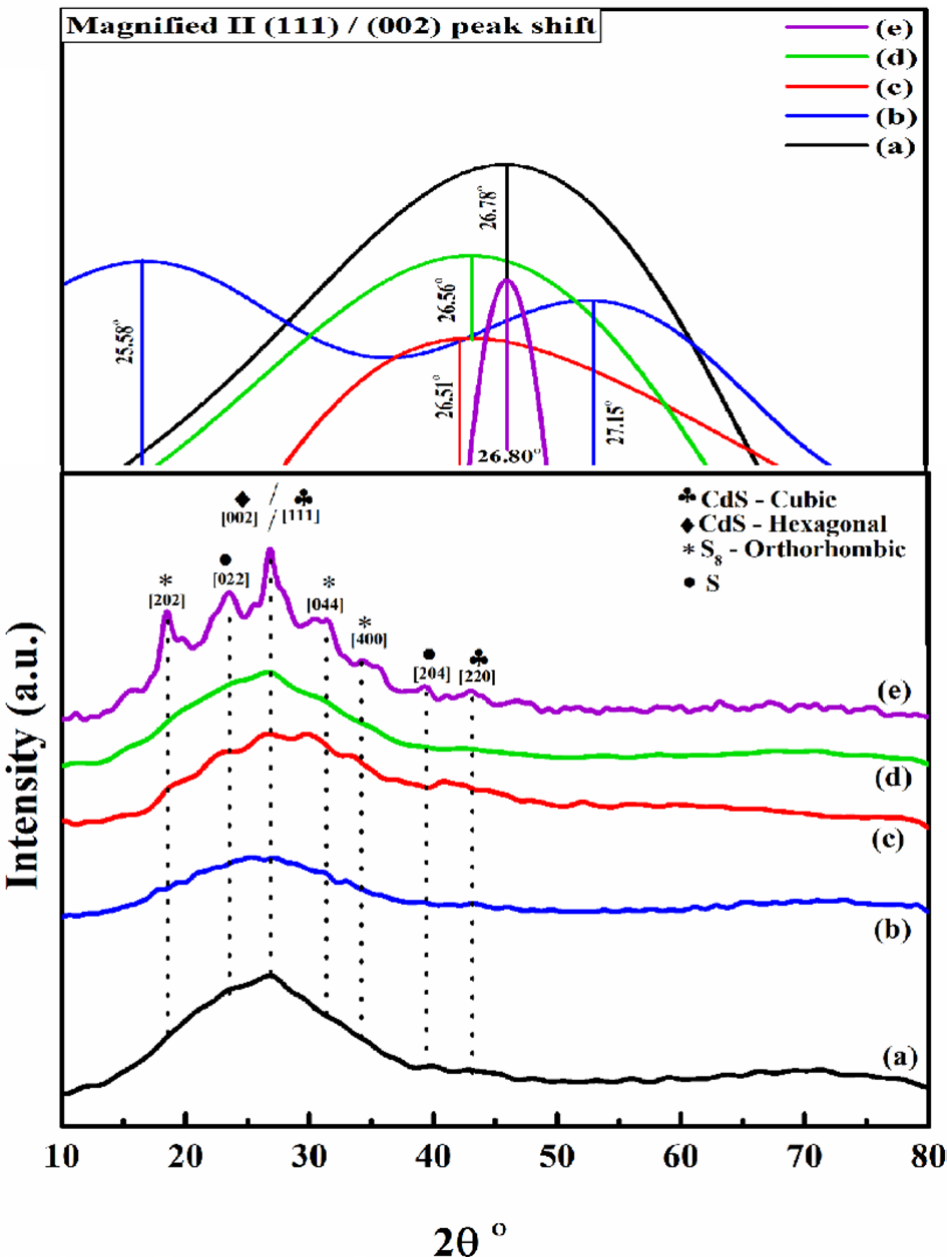
X-ray diffraction patterns of: (**a**) CdS; (**b**) CdS + Salvia dye; (**c**) CdS + Ag + Salvia dye; (**d**) CdS + MPA + Salvia dye, (**e**) CdS + mix all.

X-ray diffraction is employed to study the influence of doping on the crystallization behavior of the investigated thin films^38^. The films were scanned from 10° to 80°. Figure 5 shows that, the XRD patterns of basic CdS and doped CdS thin films. Based on the deposition conditions, CdS film primarily forms cubic and hexagonal phases. Furthermore, it is difficult to identify the crystal structure of a CdS thin film, whether it is predominantly hexagonal, essentially cubic, or a mixture of both, because both film phases have the same XRD diffraction peak angles^39^.

The XRD analyses of the samples indicated the presence of many strong diffraction peaks at 26.7°, and 43.3° which were attributed to the (111), and (220) lattice plans, respectively. These matched the cubic phase of the CdS (JCPDS 00-002-0454) in all the samples, which established the presence of CdS as the base in the above samples. Furthermore, the XRD analyses of the crystalline structure of the CdS thin films indicated the presence of a strong peak at 26.84°, indexed to (002), for the hexagonal CdS planes (JCPDS 01-080-0006).

Results indicate that there is no separate Salvia dye peak suggesting that CdS synthesis is accomplished by reactions without structural alteration. However, from Fig. 5b the introducing the dye has affected by shifting positioned at 2θ = 25.58°, oriented along with the (002), directions and are in good agreement with the (JCPDS 03-065-3414), suggesting the hexagonal form only. Figure 5c showed the mixing of both Ag with Salvia dye which displayed random structure shifting positioned at 2θ = 26.51°, oriented along with the (002), directions and are in good agreement with the (JCPDS-00-041-1049), suggesting the hexagonal phase only. This result has been expected due to the impact of Ag firstly as consider here as impurities that decline of the lattice constant and the crystalline plane distance. Secondly, Salvia dye here was hinder interaction also. Figure 5d show another mixing that happened between MPA and Salvia dye. Compare with Fig. 5c it can observe that the mixing is positioned less random orientation with the (JCPDS-00-041-1049). The most inhomogeneous structure from all samples is related to Fig. 5e. The presence of a large number of peaks indicates the polycrystalline films, because of the continuous growth of CdS along the direction with the interference of each additive (which in this case added all together in the same reaction) peaks were relatively weak compared to those in the other cases. It can consider that CdS appearing in both phases cubic (JCPDS 01-080-0019) and hexagonal (JCPDS 00-041-1049), were indications of their good crystalline nature in terms of formation of CdS. Despite that, two different phases appeared related to unreacted sulphur. It can explain that by back to the conditions of reaction. As in this case CdS formation almost finished, accordingly there is no sufficient CdSO_4_ to reacted with MPA that was as the second source of sulphur, that has been adding after 30 min from reaction started. So, the excess of sulphur formed in two phases of sulphur as S (JCPDS-01-074-2108) and S_8_ (JCPDS-01-085-0799).

Bragg’s law is used to determine the crystallinity of the produced film in terms of lattice constant, phase, strain, and defect densities using XRD spectrum data. In a cubic structure, structural parameters are computed using Bragg’s law and Vegard’s law;

2 $${\text{Bragg's law:}}\,\, n\lambda = 2d\,\text{sin}\theta,$$

3 $${\text{Vegard's law:}}\,\, a_{cubic}={d}_{hkl}({{h}_{2}+ {k}_{2}+ {l}_{2})}^\frac{1}{2},$$

where, n is the diffraction order, λ is the wavelength of the incident X-ray, θ is the diffraction angle and d is inter- planar spacing^40^. Consequently, the out and in plane lattice constant c, and a related to hexagonal unit cell can be estimated by;

4 $${a}_{hex}={\left(\frac{1}{2}\right)}^{1/2} {a}_{cubic },$$

5 $${c}_{hex}={\left(\frac{4}{3}\right)}^{1/2} {a}_{cubic}.$$

The Debye–Scherrer equation is used to compute the crystallite size (D_hkl_) using the strongest peak^41^.

6 $$D=\frac{0.94\lambda }{{\beta \, \cos \,\theta }} ,$$

where D is the mean crystallite size, λ = 1.5408 Å is the X-ray wavelength, θ is the Bragg diffraction angle and β is the full width at half maximum (FWHM) of the diffraction peak, respectively. Besides crystallite size, strain in thin films is described as the disarrangement of the lattice formed throughout deposition and is dependent on the deposition conditions. Accordingly, lower strain value indicates better crystallinity. The strain is calculated using the following equation^42^:

7 $$\upvarepsilon =\frac{\beta\, }{4\,\text{ tan}\theta }.$$

Dislocation density is investigated through Williamson and Smallman’s relation;

8 $$\updelta =\frac{n}{{D}^{2}}.$$

In polycrystalline crystals, D attributes to the crystallite size, or the intermediate diameter of each crystal orientation, and crystallite size reduction increases the lattice mismatch^43^. Table 2 summarizes the calculated parameters.

**Table 2:** Structural parameters of CdS thin films having different cases.

Sample code	hkl	2θ°	FWHM (β)	a (A°)	c (A°)	d_hkl_ (nm)	D (nm)	ε [%]	δ (× 10^–4^)
(a) Basic CdS	(111)/(002)	26.78°	0.620	5.82/4.12	5.82/6.68	0.332	13.3	1.136	56.6
(b) CdS + Salvia dye	(002)	25.58°	1.047	4.13	6.73	0.348	7.8	2.014	164
(c) CdS + Ag + Salvia dye	(002)	26.51°	0.203	4.14	6.71	0.333	41.9	0.373	5.6
(d) CdS + Salvia dye + MPA	(002)	26.56°	0.300	4.14	6.71	0.335	28.0	0.554	12.7
(e) CdS + Ag + Salvia dye + MPA	(111)/(002)	26.80°	0.341	5.81, 4.14	5.81, 6.71	0.332	26.6	0.576	14.1

As can be observed, the values of crystallite sizes are located in the nanometre range (7.8–41.9) nm, suggesting that the polycrystalline CdS films are made up of nanocrystal particles.

### Morphological analysis

**Figure 6:**
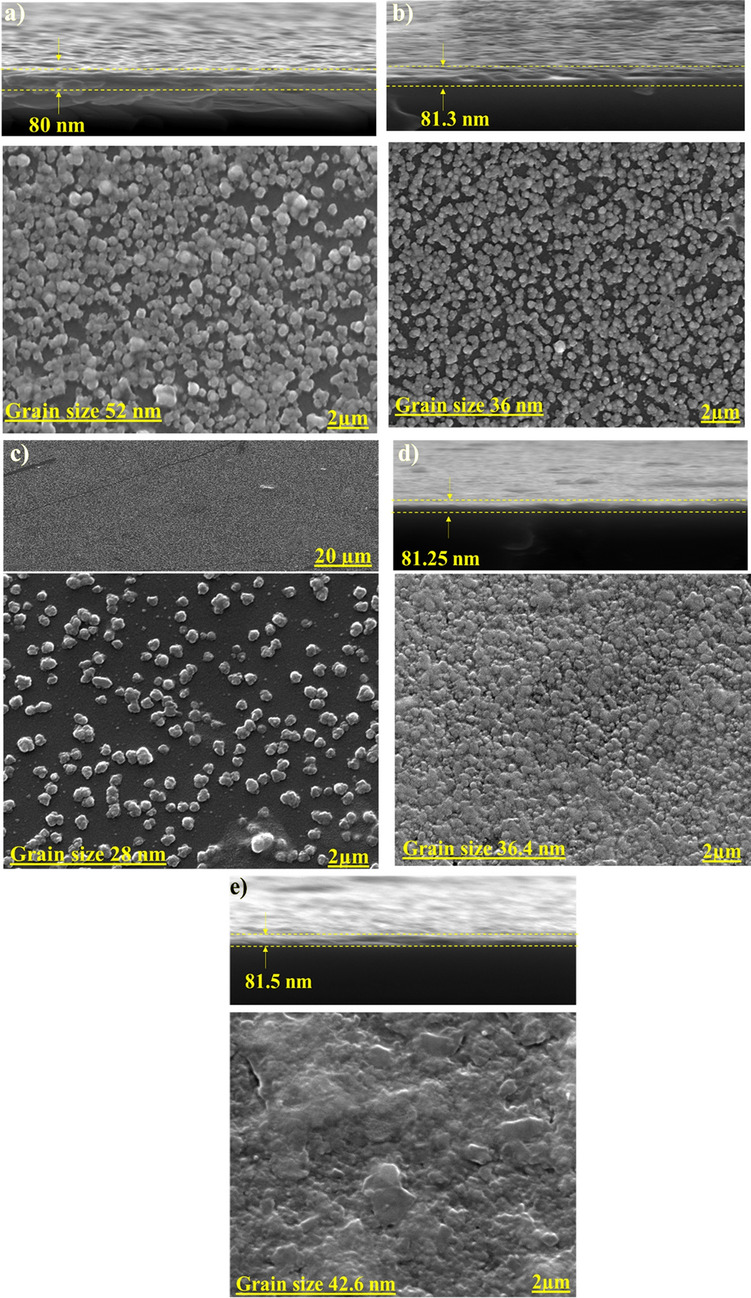
FESEM images, cross section and EDX for; (**a**) CdS; (**b**) CdS + Salvia dye; (**c**) CdS + Ag + Salvia dye; (**d**) CdS + MPA + Salvia dye, (**e**) CdS + mix all.

It has been revealed that the morphology of CdS films has a considerable impact on their overall properties. This was especially noted in the solar cells, wherein the surface roughness and grain boundaries affected the recombination in the films. Hence, it was concluded that the operational parameters of the photovoltaic devices could be affected due to the surface of morphology and the presence of any impurities on the surface^44^. FESEM images for the CdS thin films has been revealed in Fig. 6.

These micrographs demonstrate that films obtained have good coverage with less pinholes or cracks. In (CdS) sample, the fine particles collect and represent the entire surface of the substrate continuously contributing to a homogenous layer Fig. 6a. This may be due to the simultaneous occurrence of both the ion-by-ion mechanism and the cluster-by-cluster mechanism on the surface of the substrate^45^. Figure 6b showed consistent and regular, with better coverage compared with previous sample. This is most likely because of the colloidal particles produced in the solvent and adsorbed in the film. In addition, Ag-doping with Salvia dye causing tension in the CdS matrix is linked to the defects in the film surface, also evident in the change in intensity in the XRD pattern. No fractures or pinholes on the surface are found in the superficial morphology, Fig. 6c. While the surface morphology of CdS film after mixing both MPA and Salvia dye reveals a great modification on the surface structure of CdS films. The grain size was rather standardized and the substrate was fully covered with CdS film, which could also serve as a compact layer to prevent the current from leaking. The morphology of the CdS thin films is changed from granular structures to more compact and dense nanoparticles, with a variation in the composition ratio as seen from Fig. 6e. Even so, certain impurities were found due to film surface contamination. The sample in this case has an architecture with different layers. The film is defined by a cluster structure and has irregular particles with a smooth cut. The further various types of materials are in a solution, the more aggregates amounts. The impact of multiple compositions in the reaction contributes to the existence of aggregates with secondary nucleation and film growth^46^. Furthermore, numerous methods for estimating the diffraction profile, such as the Scherer formula, are effective, and while they can approximate the precise value of crystallite size, they are not comparable. The size of the crystalline domains is determined by XRD, whereas physical grains are revealed by FESEM. A single grain may include multiple domains with varying orientations. As a result, the size measured by FESEM will be either bigger or equal to the size predicted by XRD in the case of perfect grains. As a result, FESEM calculates grain size as an average value, whereas the Scherrer technique uses diffraction data from a single plane at specified 2θ and FWHM to measures crystallite size. The grain size varying from 52 to 28 nm, as a higher and lower values for basic CdS and CdS + Ag + Salvia dye respectively.

**Figure 7:**
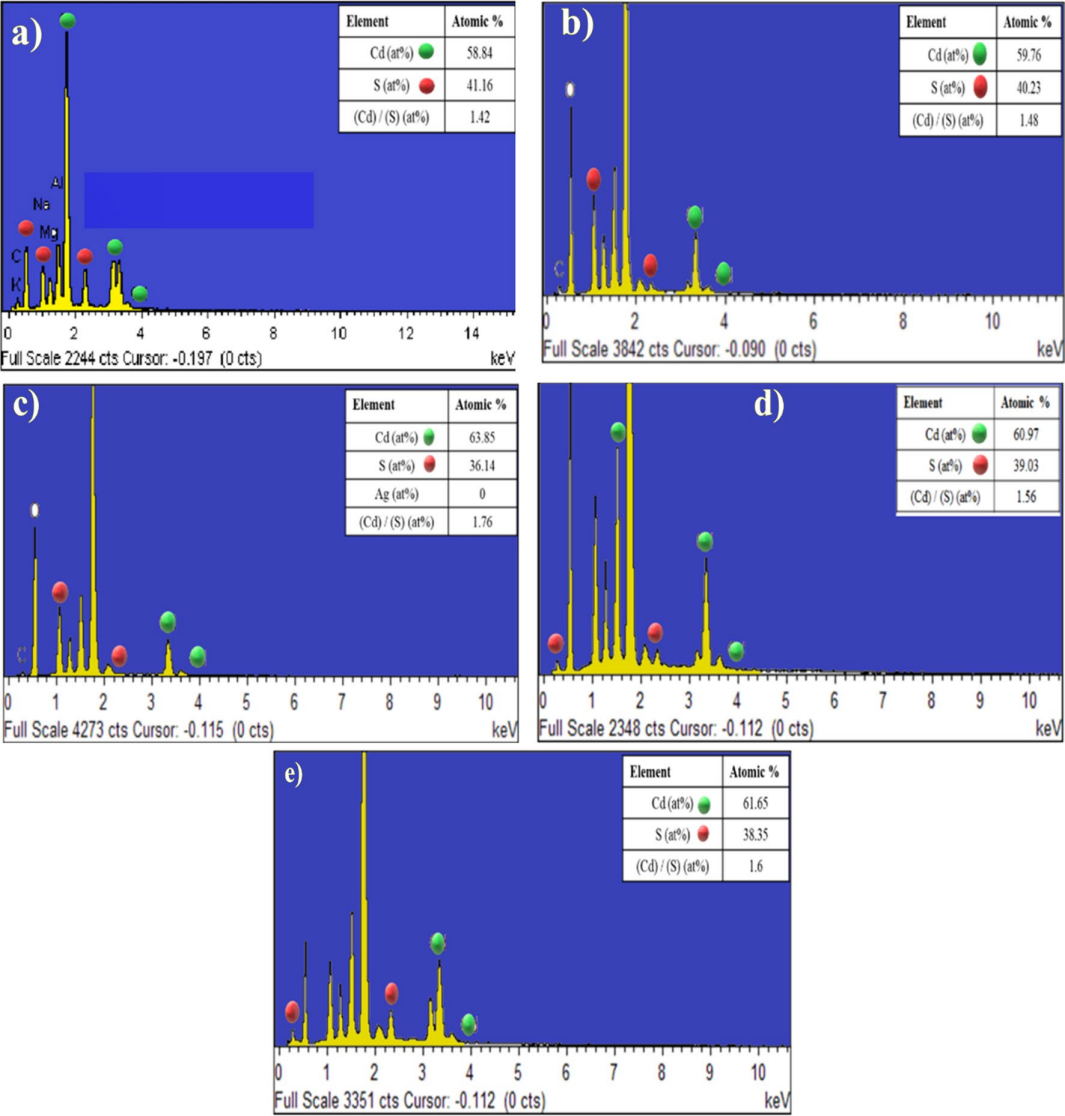
EDX for; (**a**) CdS; (**b**) CdS + Salvia dye; (**c**) CdS + Ag + Salvia dye; (**d**) CdS + MPA + Salvia dye, (**e**) CdS + mix all.

We acquired the EDX spectra of all prepared samples for determining the chemical composition of the film surface^47^. It was noted that the sulphur ions were homogeneously distributed in the sample, which indicated that the sulphur-containing ligand was capped onto the surface in small quantities, Fig. 7. The stoichiometry of CdS films is determined by the Cd/S ratio, which varies from case to case. Cd^2+^ ions are released by CdSO_4_, which is considered to be the most appropriate source of Cd due to the highest deposition rate when compared to other Cd sources, while S^2−^ ions are supplied by the CBD process’s decomposition of thiourea. CdS thin film with rich Cd, have a wider bandgap range, superior grain structure, and excellent mobility.

### Topological studies (AFM)

**Figure 8:**
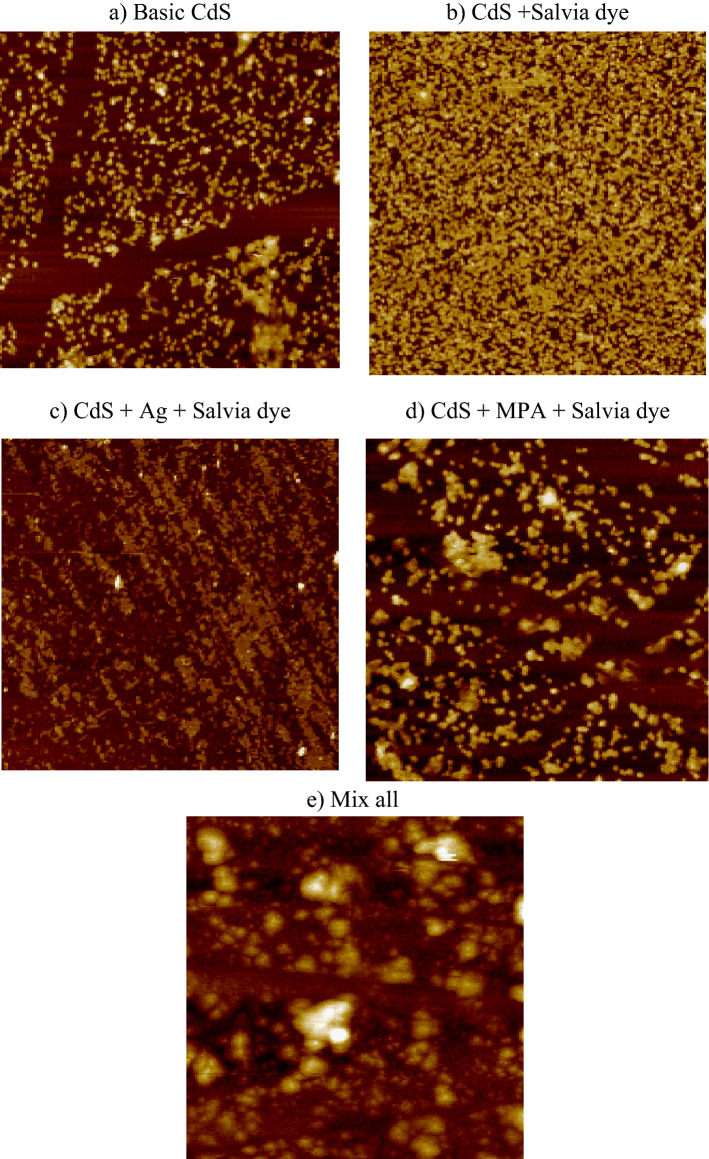
2D AFM images for different CdS additives.

**Figure 9:**
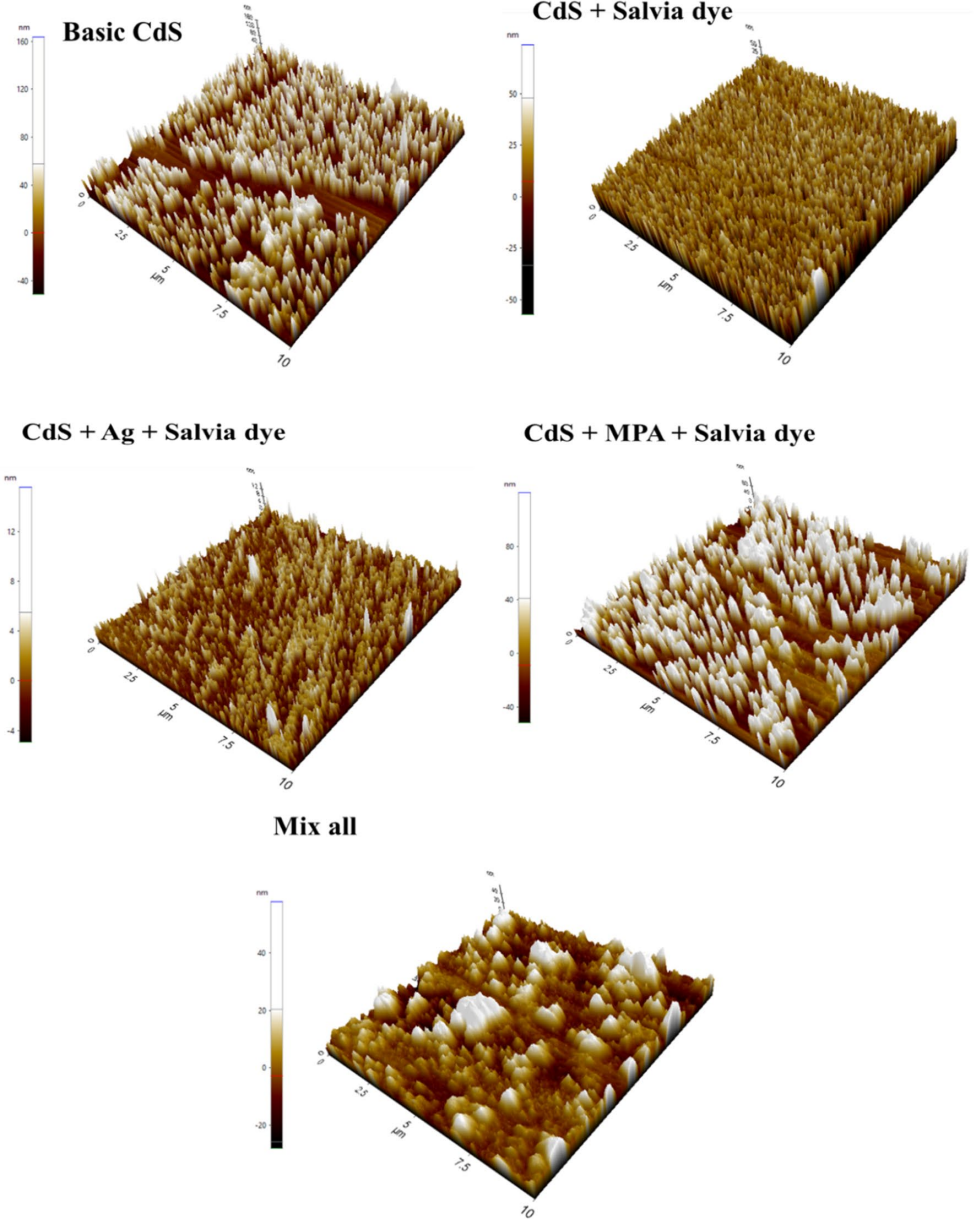
3D AFM images (10 × 10 μm) for different CdS additives.

The surface analysis gives unique insights into the surface topological properties of CBD-grown CdS thin films by using the AFM technique^48^. This technique provides digital images that enable surface features to be calculated quantitatively, such as root mean square (RMS), and image analysis from various perspectives, including three-dimensional simulation. Part of the purpose of using AFM is to analyze the contribution of the incorporation of various materials to film quality. The 2-D and 3-D image topography was used by AFM to show CdS thin film samples topography as a different growth approach, Figs. 8 and 9. To calculate the total average surface roughness, S_a_, and observe particle aggregation, surface topology mapping was performed over 10 μm × 10 μm scan areas.

The surface of all samples reveals a distinct distribution of grain with a uniform granular topography. The surfaces consist of nano-sized grains and the average roughness of the deposited CdS thin films was (1.6–24.3) nm, which indicates the formation of smooth and well-connected grains on the film, Table 3. In some instances, small white spots across some clusters have been found from both FESEM images and AFM images. The formation of white spots has been confirmed and corresponds to unreacted sulphur^49^. The higher value of roughness was attributed to the presence of hill height due to a significant number of nucleation and large grain growth^50^. The value of the skewness was positive as well as small, close to zero, except in sample (b) due to the influence of the Salvia dye, which reflects that the surface has fewer peaks than the valleys and that the height distribution is far from symmetrical.

**Table 3:** AFM analysis.

Samples	Average roughness [S_a_] (nm)	Root mean square [RMS] (nm)	Surface skewness [S_sk_]	Surface Kurtosis [S_ku_]	Top Ten height [S_z_] (nm)
Basic CdS	24.3	29.3	1.08	3.11	215
CdS + Salvia dye	20.4	24	− 0.555	2.14	130
CdS + Ag + Salvia dye	1.6	1.9	1.11	5.02	20.5
CdS + MPA + Salvia dye	23.4	28.5	1.018	2.92	171
Mix all	7.8	10.4	1.16	5.27	8.58

The results indicate that the surface topography of sample Fig. 6a, a low smooth surface with an average roughness of 24.3 nm, besides a small number of summits appears sported by a low value of root mean square 29.3 nm, owing to the initial growth of CdS, which could be due to a low growth rate of CdS thin film or possibly due to the consumption of the growth solution. The properties of CdS vary based on the ion concentration and the synthesis conditions such as heat and pH; thus, any incorporation of new reaction material induces variations in the effects of AFM. A low influence on RMS was found for all samples collected by mixing with various additives. Sample Fig. 8b indicates a sharp acclivity of roughness and RMS to 22 nm, attributed to a rise in the concentration of growth solution after the addition of Salvia dye, which may be due to a decline in the feedstock of incoming on the substratum, with an increase in the concentration of growth solution. The two-dimensional AFM images show the morphology of the pinhole-free surface. AFM image for sample Fig. 6c reveals an irregular film surface with no cracking and thick morphology, showing that the CdS film forming process is attributable to the deposition of clusters-by-clusters at the beginning. AFM studies showed that, compared to other films, the surface roughness was comparatively minimal. The low roughness means the consistency of the film is reasonably good. However, the small particles (RMS) are eventually transformed into large particles of 28.5 nm RMS with further addition of MPA to the Salvia dye sample Fig. 8d. Particles may be here because of particles of Salvia dye surrounded by nanostructures of CdS. The roughness of basic CdS is still evaluated higher than in all cases we described above. RMS roughness values vary with the various materials, especially for combining all samples Fig. 8e. Also, it may see scattered spherical-like structures composed of micro-particle clusters surrounding the initial 20-min compact layer. These regular spherical structures reveal a compact nucleus and a less compact shell. Growth tends to be a mixed-mode in this scenario, in which the film initially nucleates in two dimensions and then gradually turns into three-dimensional growths. Micrographs of surfaces deposited indicate an increasing degree of CdS grain coalescence and vertical growth.

### Hall measurement

**Figure 10:**
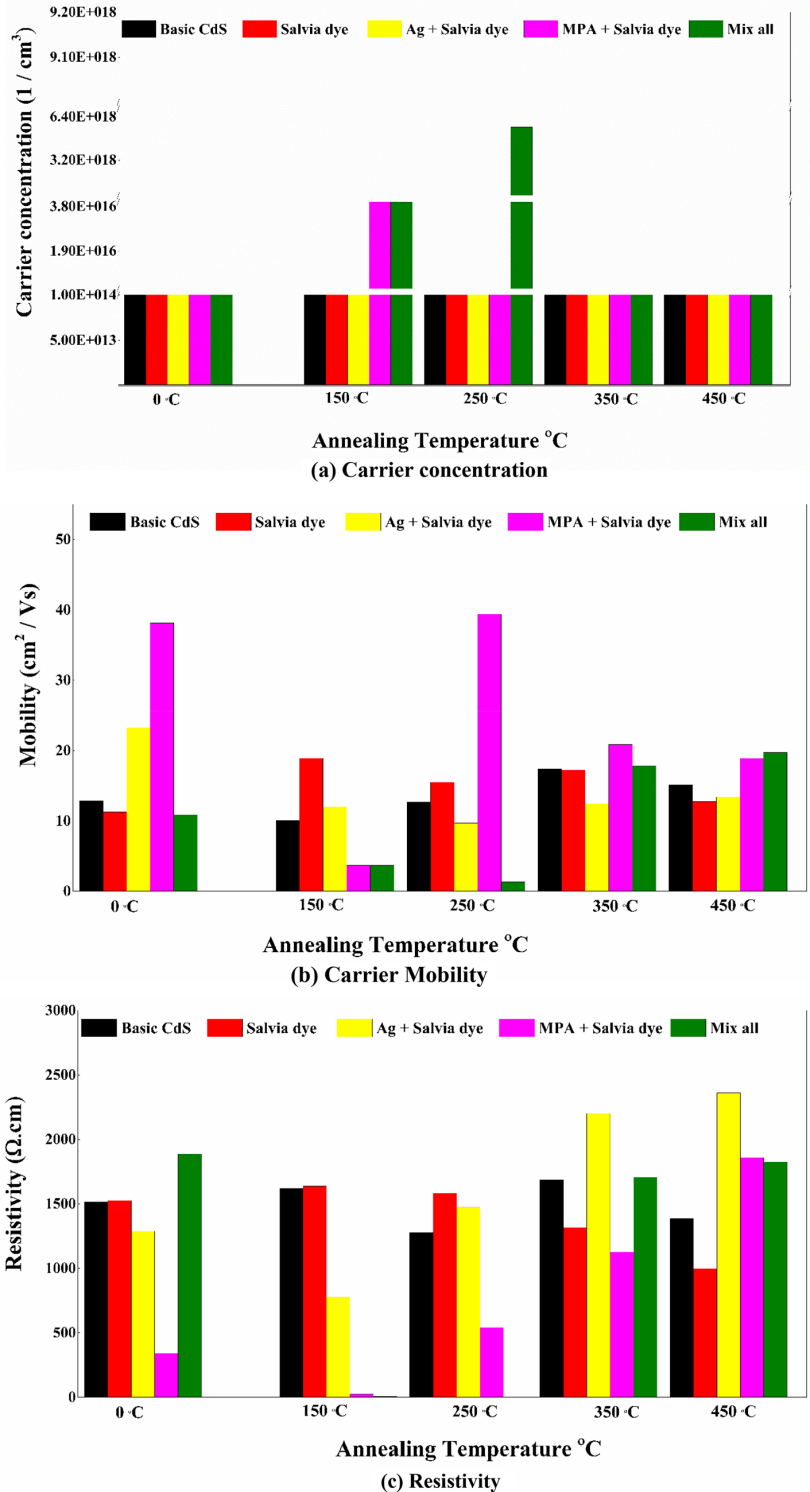
Electrical properties for various CdS thin film samples.

Since CdS thin films are used in the form of a buffer layer in the thin solar cells, their higher conductivity can help in effectively separating the generated charge carriers during the photovoltaic energy conversion. This subsequently increases solar cell efficiency. To investigate the maximal changes occurring in the properties of the material, we selected the annealing temperatures between 150 and 450 °C with 10 min as the annealing time. This study was similar to that conducted by Akbarnejad et al., who selected varying annealing temperatures ranging between 300 and 500 °C^51^. The CdS samples undergoing heat-treatment showed a higher conductivity value in comparison to the non-treated CdS films. We verified the n-type conducting property of the CdS thin films by determining the negative values of the Hall coefficient for all the samples. Figure 10 presents the electrical characteristics of the samples under varying annealing temperatures.

For example, a shift from the metastable cubic to the stable hexagonal phase of CdS has been shown to have produced a continuous change in structural, optical, and electrical properties, as seen in electrical resistivity. Whereas, the mixing dye was varied (before annealing) both carrier concentration and carrier mobility did not indicate significant changes. This may be related to the weak absorption into the lattice of organic dye and CdS, probably as a result of the dye’s poor solubility in the growing solution. Besides, the minimum ρ values, 340.71 and 1288.36 Ω cm, were achieved for (CdS + MPA + Salvia dye) and (CdS + Ag + Salvia dye). This may be due to the rich incorporation of mixing additives in the lattice, possibly because of the good solubility of the doping salts in the solution for growth. When the MPA concentration was present, the resistivity of the doped films changed significantly.

**Figure 11:**
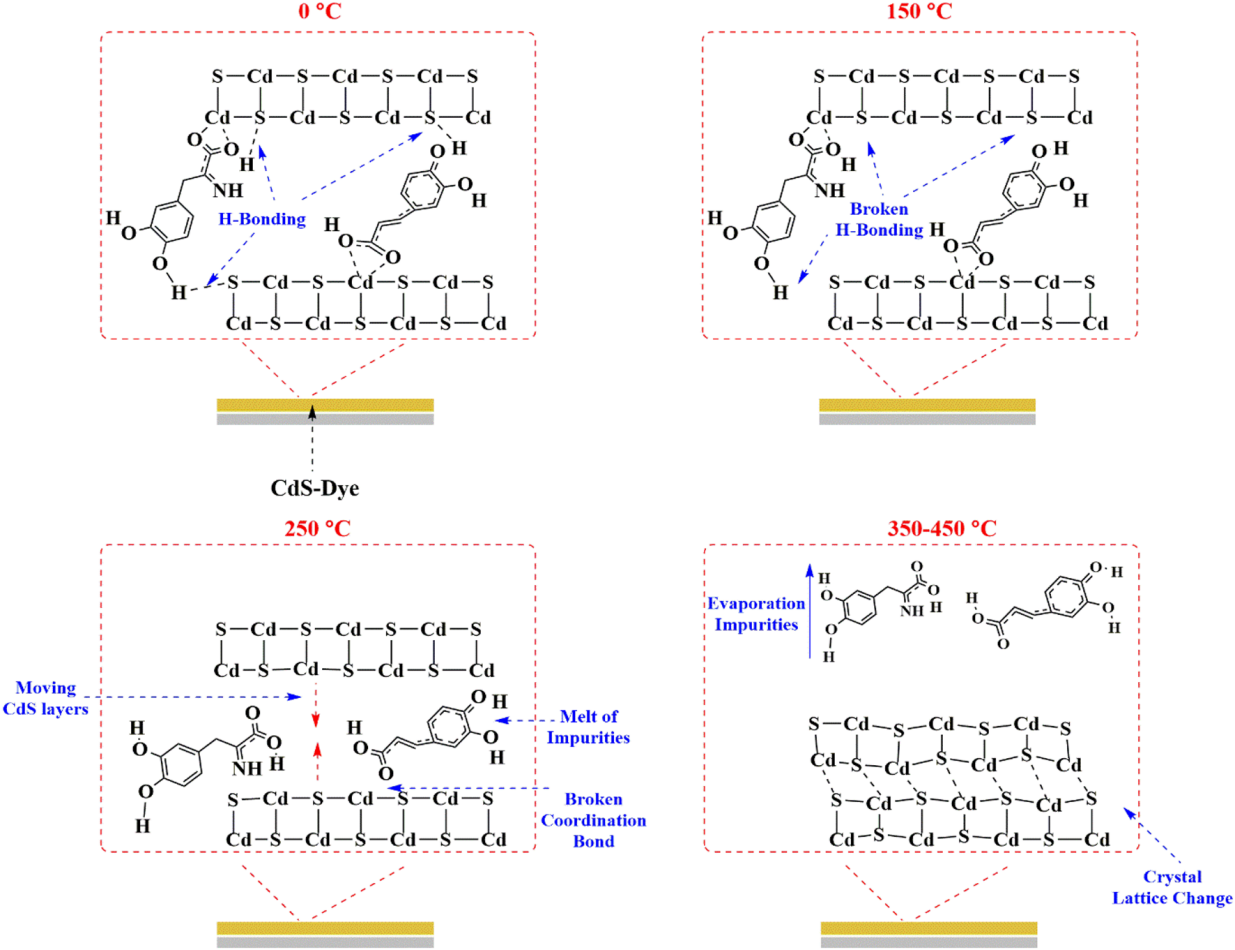
Remove impurities and changes of crystal structure that occur during the annealing process.

Properties such as a narrowing of the bandgap, a reduction in the grain boundary density and density of dislocation, and an increase in the carrier concentration led to an enhancement in the grain size of the films^52^. Referring back to Fig. 6, which depicts the grain size of each sample, we can see that the greatest value corresponds to basic CdS, while the second sample represents CdS + mix all. We can explain the decrease in optical scattering as a result of the densification of grains followed by grain growth and the reduction of grain boundary density in the case of all-additive mixes. In contrast, the increase in carrier concentration has not been seen for basic CdS produced by the traditional approach (without the addition of any additives), despite the fact that its grain size is regarded as the greatest. Here also, the enhanced film conductivity in relation to the film structure may be associated with the improved film crystallinity at annealing temperatures, Fig. 11.

After annealing, this rise in film crystallinity is apparent by analysing the XRD trends, which indicates the gradual increase in sharpness of the CdS peak after a particular additive. Moreover, the possibility that the hexagonal phase of CdS is becoming progressively common is also a contributing factor.

**Figure 12:**
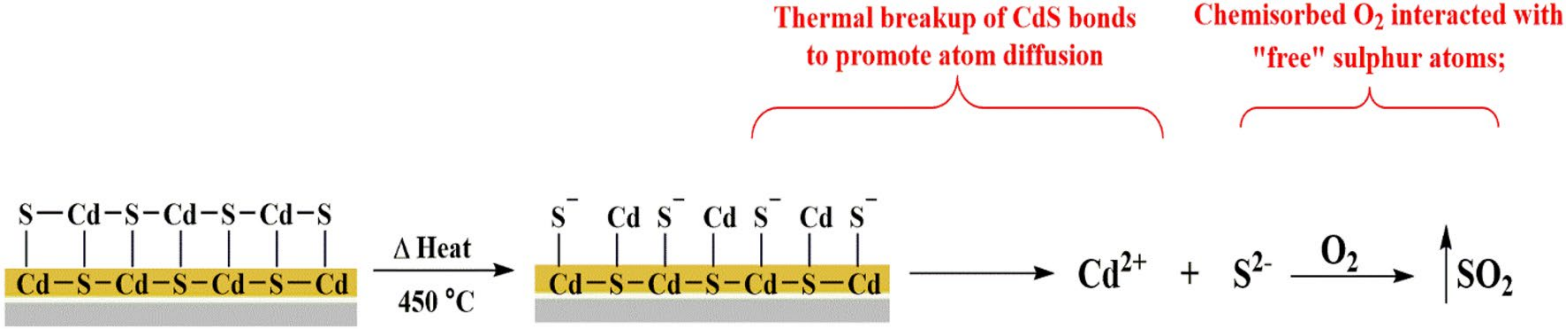
The side reaction undergoes at the CdS surface.

Indeed, when the air annealing has been introduced in films, the electrical properties are enhanced. Since conducting CdO phases predominate and with a smaller extent in CdSO_4_ phases, the film has a higher conductivity at 250 °C. Due to chemically created extra trapping centres in the film, these film impurities exhibit high current and slow photoresponse properties. The high conductivity of chemically produced CdS films after air annealing makes them an excellent buffer layer for heterojunction solar cells. This allows reducing the resistance series of the structure of a layered solar cell. At mix all samples with an annealing temperature 250 °C, the resistivity has been recorded as (0.83 Ω cm) which is considered as the minimum value in the literature for CdS thin films prepared via CBD method. These films have higher optical transmission and a smaller grain size. But further annealing at ≤ 350 °C populated more CdS ions thus decreasing film conductivity, this can be explained because the melting point for CdS is 980 °C, and as a rule of thumb, at 1/3 of this value around 326 °C, the crystallinity of CdS has been deteriorated. This led to the current compound breaking down into its constituent atoms that disperse within and outside the CdS matrix and are free to interact. According to that, the cadmium and/or sulphur atoms undergo “out-diffusion”. As a consequence, the resistivity increased. Potential reaction pathways arising at temperatures of T > 450 °C include, as shown below in Fig. 12:

## Proposed mechanism

**Figure 13:**
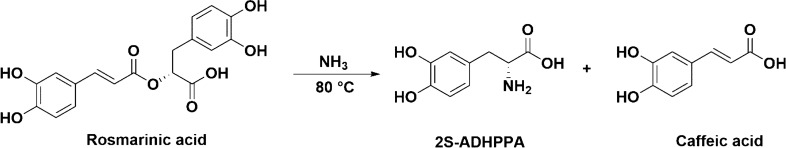
Hydrolysis of the Rosmarinic acid.

Aqueous extracts *S. officinalis* is rich in flavonoids, mostly Rosmarinic acid^8^. Rosmarinic acid was hydrolysed in the alkaline solution into (2S)-2-amino-3-(3,4-dihydroxyphenyl) (114C) propanoic acid (2S-ADHPPA) and Caffeic acid^53^, as shown in Fig. 13.

**Figure 14:**
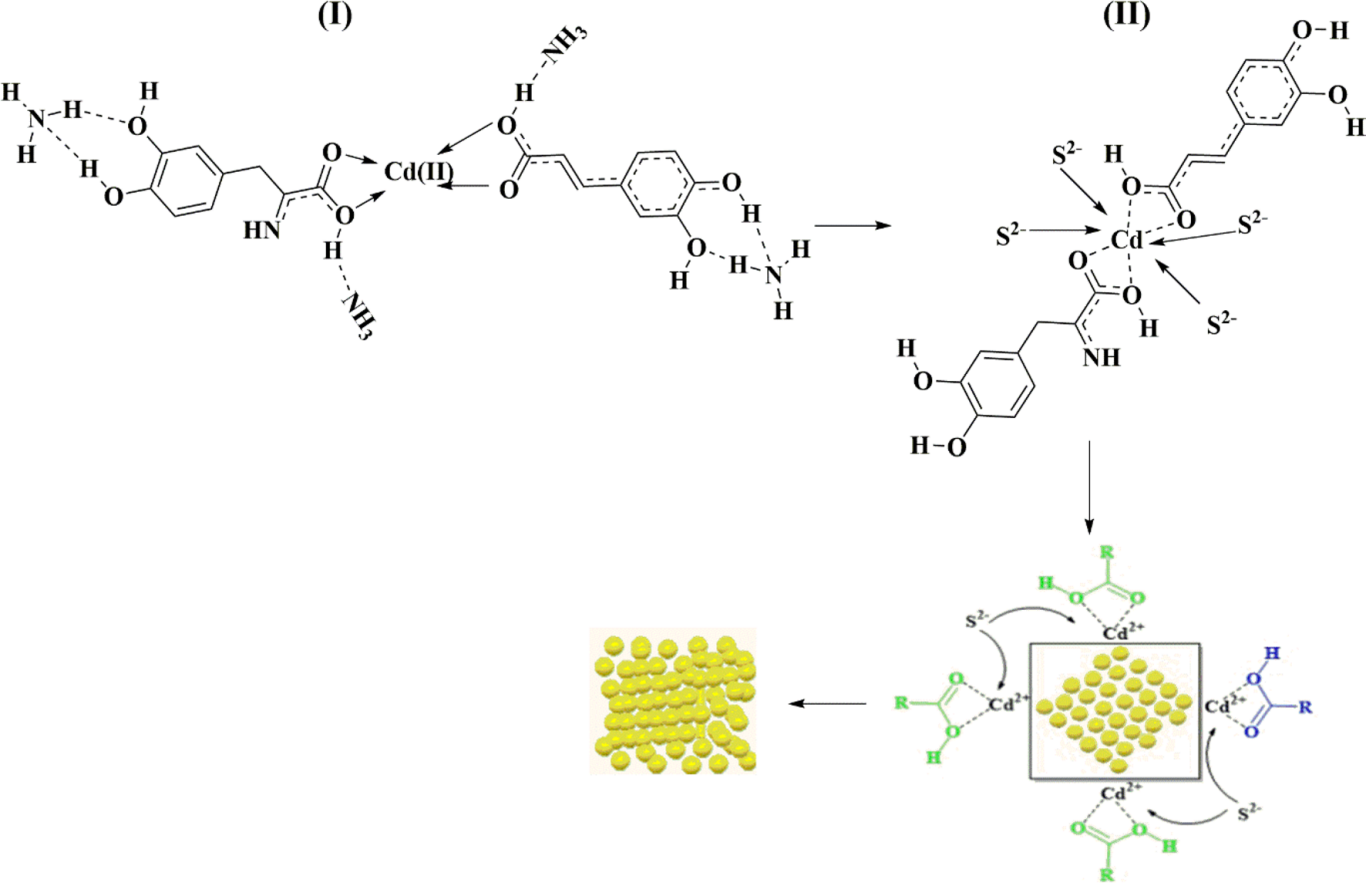
The proposed mechanism of the rule of the dye solution in CdS formation.

These two compounds have the advantage of having active sites of bi-chelate carboxylic groups which can be coordinated with cadmium ions in the solution. Each group has two oxygen atoms that are capable of contacting these ions easily and without a steric hindrance. The affinity of the active site in 2S-ADHPPA is enhanced by the introduction of the primary amine group –NH_2_ on an alpha-carbon (α-C) providing sufficient electron density towards cadmium ions Cd^2+^ to form additional stable intermediate complexes within the solution. While the affinity of the active site in caffeic acid is enhanced by hydroxyl groups on the benzene ring after deprotonation by a strong NH_3_ base as shown in Fig. 14I. It should be noted that the mechanisms that occur during the CdS thin film formation period are the ion-by-ion mechanism, the molecule-by-molecule mechanism, and the cluster-by-cluster mechanism. The ion-by-ion mechanism is responsible for the formation of CdS molecules in solution, as well as for the growth of clusters and the filling of voids on the surface of CdS film. The ion-by-ion mechanism has a long lifespan until the reaction is over, due to the continue of releasing ions of cadmium and sulphur in the solution. While the molecule-by-molecule mechanism has a short life span, it may be responsible for the formation of the cluster nucleus in the solution, in addition to being responsible for the formation of the hexagonal phase of CdS^54–57^. The amount of *S. officinalis* solution was added to the reaction beaker after 20 min of the life of the reaction. Our observation shows that CdS layer at around 17 min becomes homogeneous, continuous, and crystalline CdS layers covering the entire substrate area, and the naked eye, we can see how the growth beaker becomes get yellow colour and then shortly become stable. These results were agreed with Sandoval and Ramírez, who investigated the early phases of CdS thin film growth during chemical deposition and revealed that the ion-by-ion growth mechanism occurs at deposition times between (15 and 18) minutes, resulting in the formation of a dense compact CdS inner layer^29^. The addition resulted in the reactivation of the molecule-by-molecule mechanism by the formation of intermediate complexes [2S-ADHPPA-Cd-Caffeic]^2+^ in cadmium ion solution. Thus, free sulphur ions attack these complexes and form CdS molecules in the solution. On the other hand, these intermediate complexes may be attracted to the surface and interfere with its layers, either by electrostatic forces with a surface that contains cadmium ends or by hydrogen bonds with a surface that also contains sulphur ends, as shown in Fig. 14II.

**Figure 15:**
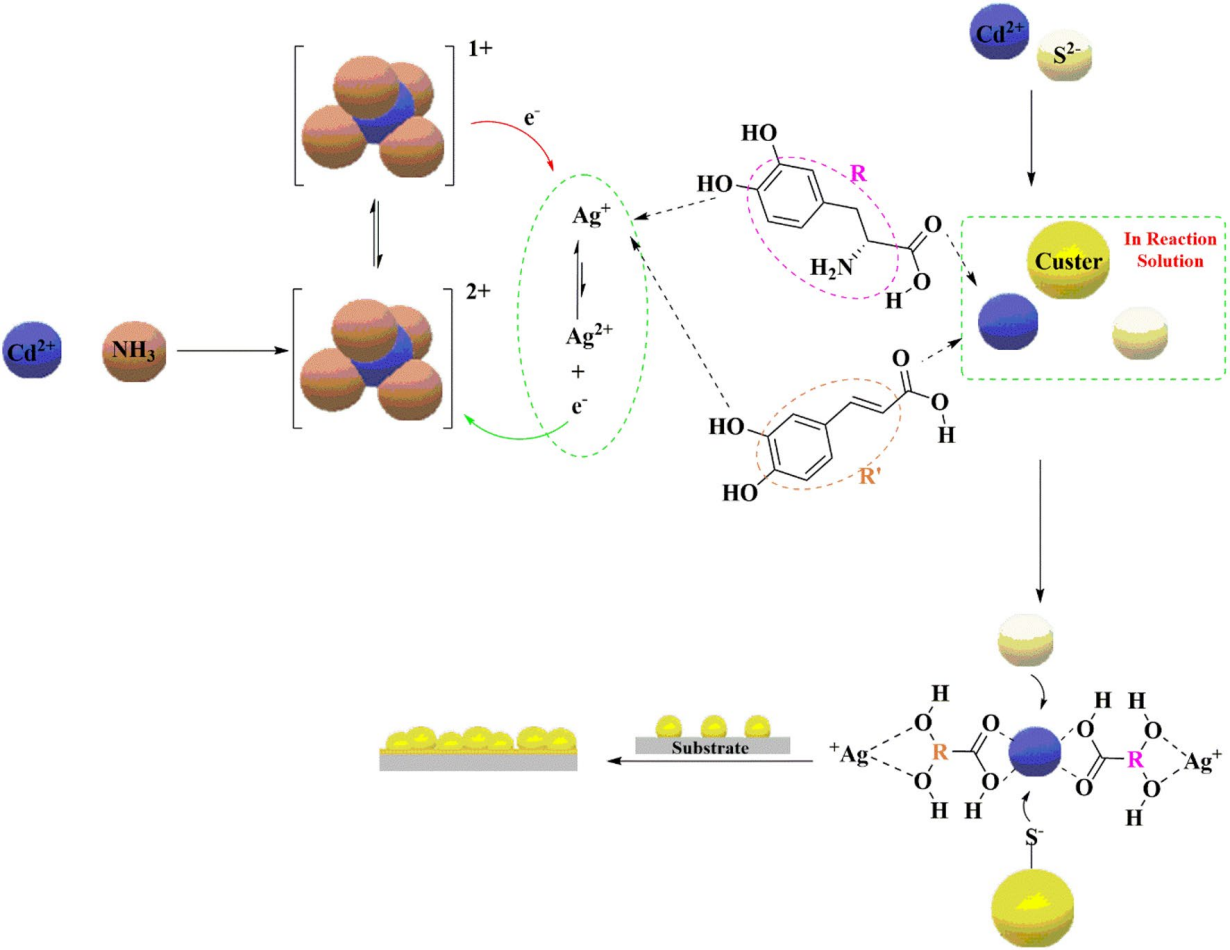
The rule of 2S-ADHPPA and caffeic acid compounds to inhibit the effect of Ag ions.

In the presence of Ag(I) and *S. officinalis*, rosmarinic acid was resistant to the effect of Ag(I) ions on the formation of CdS. Rosmarinic acids can actively chelate metal ions and reduce them to nanoparticles^58^. This capacity to generate nanoparticles is a result of their abundance of essential functional groups, including several hydroxyl groups and carbonyl moieties. As a result, the high concentration of rosmarinic acids in dentatus water extract has facilitated the reduction of Ag(I) to Ag^0^^59^. In its compounds, cadmium displays almost exclusively a + 2 oxidation state, as in the colourless Cd(II) ion, which forms a number of stable ions. Where cadmium is in the + 1 oxidation state, it is unstable in water and immediately disproportionate to cadmium metal and Cd(II). The keto–enol tautomeric transformation of the rosmarinic acid may enable the release of reactive hydrogen atoms, which drive the reduction of Ag(I) ions. Each compound inhibits the effect of two Ag^+^ ions on benzene rings by hydroxyl groups. The hydroxyl groups give the Ag^+^ electron a lost hydrogen atom to convert it from enol to ketone, as shown in Fig. 15. Ag^0^ forms and precipitates in the reaction solution without interference with the CdS layers formed.

**Figure 16:**
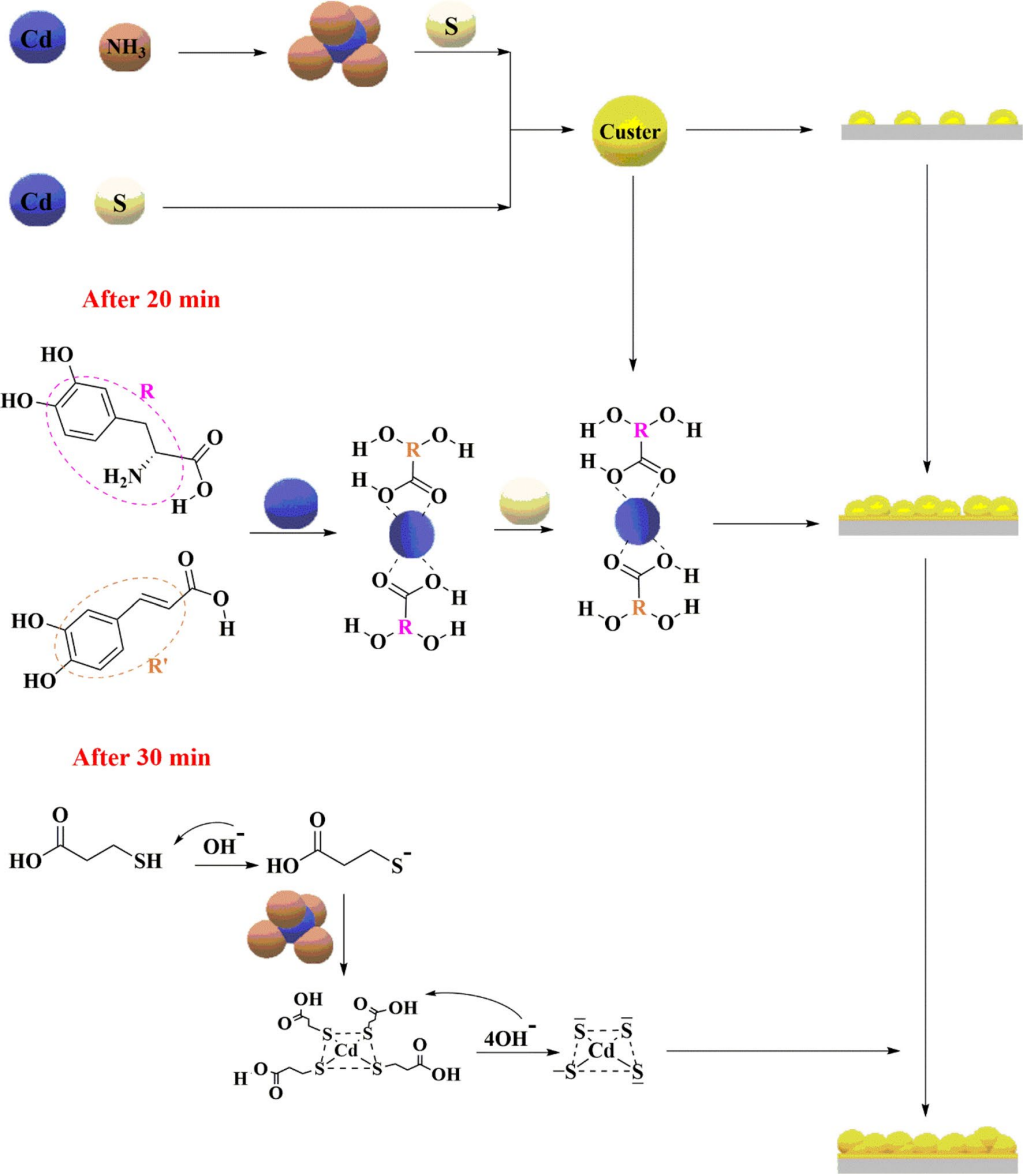
The rule of 2S-ADHPPA and caffeic acid compounds to form CdS in the presence of MPA.

The mixture of CdS reactions started with the addition of cadmium salt with 20% ammonium hydroxide as a strong base and 80% water. Twenty minutes after the reaction began, the *S. officinalis* has been added to the reaction solution. The rosmarinic acid functional groups were coordinated with cadmium ions to form intermediate tetrahedral complexes. This configuration allowed the formation of a layer of CdS compounds with tetrahedral geometry. The addition of the MPA was completed after thirty minutes of reaction. This MPA worked in direct coordination with cadmium ions through the thiol group (HOOCCH_2_CH_2_S^−^) to form an intermediate complex. This complex (HOOCCH_2_CH_2_S)_4_Cd loses its configuration and decomposes the compound into a square planar geometric CdS, as shown in Fig. 16.

**Figure 17:**
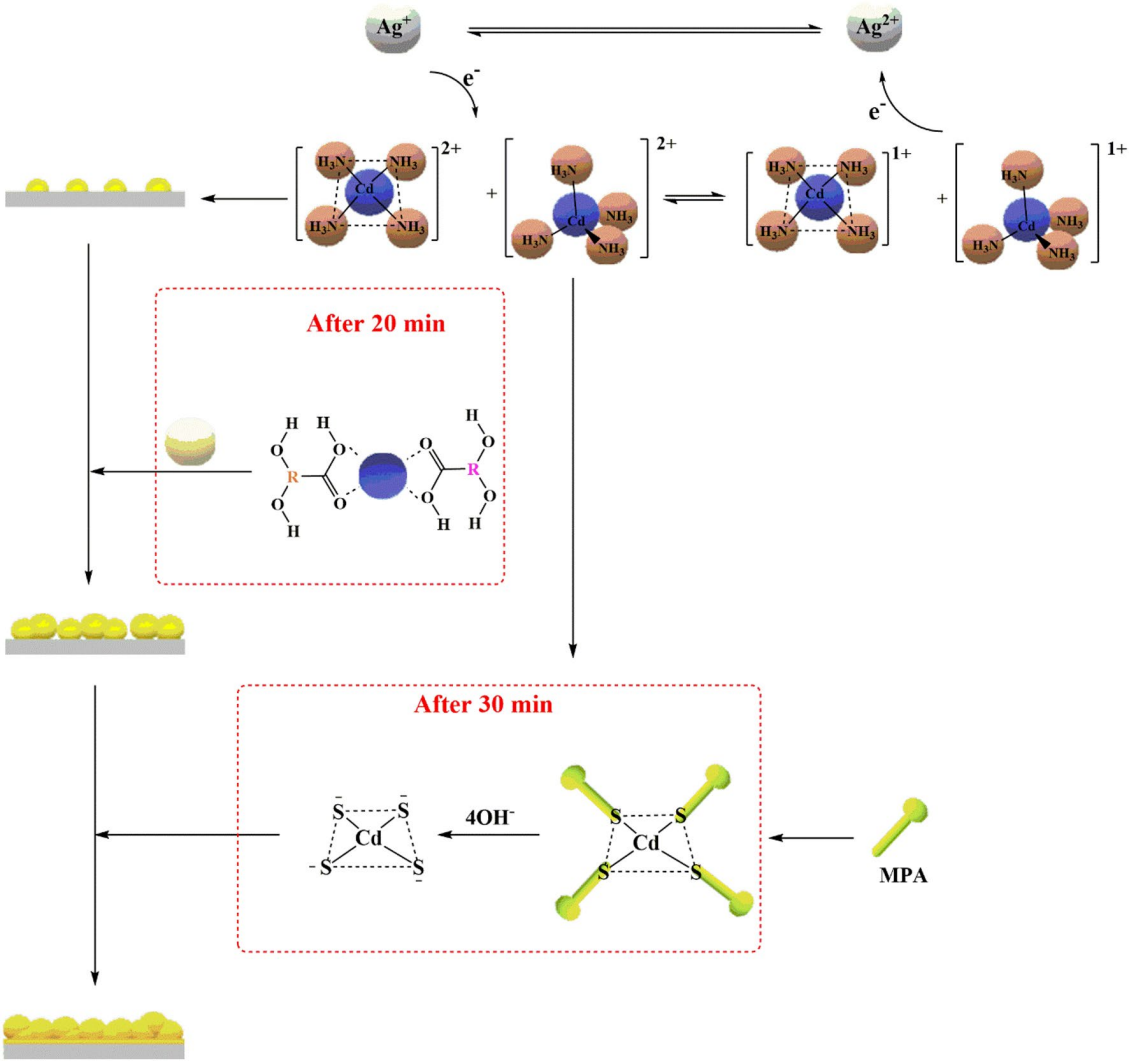
The mechanism of CdS formation according to graduate additions of Ag(I), Salvia dye, and MPA.

In the last case at Fig. 17, the participation of both rosmarinic acid and MPA with the presence of added Ag(I) ions at the beginning of the reaction. The mixture was made with the addition of cadmium salt and silver salt to 20 ml DI. The process took place with the same procedures, where the dye was added after 20 min, followed by the addition of the MPA after 30 min. This process led to the creation of S_8_ Orthorhombic and CdS products. The stepwise addition of rosmarinic acid and MPA was achieved with sufficient time to control the negative effects of silver ions and the formation of CdS layers of good properties.

## Conclusions

The growth method and growth parameters have influenced the majority of the electrical and structural properties of CdS films. In order to fabricate CdS films, a linker-assisted chemical bath deposition technique was introduced and optimized. The optical results showed a varying bandgap ranging between 2.17 and 2.5 eV. This increase in the energy bandgap for the CdS thin films was favourable for the buffer layer. XRD indicated that the CdS thin films crystallised in 2 different structural phases, i.e., cubic and hexagonal wurtzite structures, which showed a preferential orientation along (111/002) reflection plane. The crystallite sizes varied between 7.8 to 41.9 nm. Furthermore, the morphological characterisation of these films indicated that the CdS + MPA + Salvia dye sample contained the best homogeneously distributed spherical grains compared to all samples. (CdS + Ag + MPA + Salvia dye) sample after 250 °C displayed the maximal carrier concentration and the least resistivity with 5.64 × 10^18^ cm^−3^, and 0.83 Ω cm. The presence of small quantities of silver salts in the chemical solution led to the redox reaction and less formation of CdS. The silver side reactions could be prevented by the inclusion of Salvia dye or MPA or both together in the reaction, as a linker agent, helped in regulating the CdS formation. After studying the various films associated with the CdS-CBD modified technique, it can be concluded that the mixed CdS films showed a crystalline structure with some defects. Hence, the technique described in this study was preferable for acquiring a better charge carrier transport and low resistive CdS thin films acted as a buffer layer in the CZTS and CIGS photovoltaic devices. We believe that this green synthetic method will be able to capture a higher amount of light, transform the energy in light into electricity in a more efficient and effective way, and yield this approach at a lower cost than other methods now in use. In addition to that, in terms of our findings, the visions indicate that there is the potential to increase the overall manufacturing yield when tuned by adjusting the bandgap, carrier concentration, and high coverage with less pinholes by using our green synthetic strategy. In particular, if our suggested technique is implemented, there is a possibility that the related costs will be low. This is because there the potential effects of module area on the cost and performance of photovoltaic systems.

## Data Availability

The datasets used and/or analysed during the current study available from the corresponding author on reasonable request.
